# Goal attribution to inanimate moving objects by Japanese macaques (*Macaca fuscata*)

**DOI:** 10.1038/srep40033

**Published:** 2017-01-05

**Authors:** Takeshi Atsumi, Hiroki Koda, Nobuo Masataka

**Affiliations:** 1 Research Institute of National Rehabilitation Center for Persons with Disabilities, Tokorozawa, Saitama 359-8555, Japan; 2 Primate Research Institute, Kyoto University, Inuyama, Aichi 484-8506, Japan

## Abstract

Humans interpret others’ goals based on motion information, and this capacity contributes to our mental reasoning. The present study sought to determine whether Japanese macaques (*Macaca fuscata*) perceive goal-directedness in chasing events depicted by two geometric particles. In Experiment 1, two monkeys and adult humans were trained to discriminate between Chasing and Random sequences. We then introduced probe stimuli with various levels of correlation between the particle trajectories to examine whether participants performed the task using higher correlation. Participants chose stimuli with the highest correlations by chance, suggesting that correlations were not the discriminative cue. Experiment 2 examined whether participants focused on particle proximity. Participants differentiated between Chasing and Control sequences; the distance between two particles was identical in both. Results indicated that, like humans, the Japanese macaques did not use physical cues alone to perform the discrimination task and integrated the cues spontaneously. This suggests that goal attribution resulting from motion information is a widespread cognitive phenotype in primate species.

The actions and behaviours of other organisms provide information concerning a wide range of adaptive behaviours, such as preying and mating. These actions are visually associated with an external element and encoded as having potential goals. A classic psychological study indicated that agent–goal association does not require information concerning bodily features and underlies human mental reasoning^1^. This capacity is impaired in people with autism spectrum disorder (ASD), suggesting that it has an adaptive value in social communication^2^,^3^,^4^,^5^. Preverbal infants show the interpretational capacity for reasoning regarding others’ goals using motion information^6^,^7^,^8^,^9^,^10^,^11^,^12^. One study demonstrated that young infants visually preferred displays with a ‘chaser’ and a ‘chasee’ depicted by geometric figures, than those with meaningless motions^11^. Studies involving human infants and individuals with ASD have suggested that the attribution of goals to inanimate agents is an innate brain function; however, the phylogenetic origin of this attribution remains unclear.

In some previous comparative studies, non-human animals’ interpretations of animated motion have also been elucidated. One avian research revealed that newly hatched chicks (*Gallus gallus*) are visually sensitive to self-produced motion of the geometric agents^13^. Primate researches have shown that chimpanzees (*Pan troglodytes*) encode the goals of inanimate moving entities^14^,^15^, and common marmosets (*Callithrix jacchus*) showed sensitivity to the goals of a robot agent with bodily features^16^,^17^. These non-human primate researches employed the looking-time paradigm, which is frequently used in of human infancy studies. For example in chimpanzee studies, researchers followed an outstanding study on human infants^7^, in which the stimulus was a simple rectangle jumping a barrier in order to satisfy the goal of approaching a circle^14^,^15^. The researchers found that the animals looked longer at the displays if the rectangle continued jumping even after the barrier was removed, whereas they did not if the rectangle went directly to the target. In a study with an operant conditioning technique, squirrel monkeys (*Saimiri sciureus*) discriminated a goal-directed motion from another motion event with similar physical features^18^. Despite some early reports above, however, what/how visual property modulates goal attribution in non-human animals remains unclear. Here, we attempted to examine the perception of goal-directed motion patterns in another primate, Japanese macaques (*Macaca fuscata*), using a chasing discrimination task^18^. We sought to determine how each physical property observed in chasing events, such as high correlation between motion trajectories^19^,^20^,^21^,^22^ and the proximity of agents^23^, contributed to perception. We hypothesized that, if the monkeys were sensitive to the goal-directedness of agents, they would display a distinctive response to chasing motion patterns. Finally, we also hypothesized that, if the monkeys could perceive goal-directedness from inanimate entities, they would show the same response trends as do human observers.

We first assessed the effects of the correlation between agents for human perception. We calculated the degree of similarity (DoS) within each stimulus, which indicated the correlation between the trajectories of two particles^18^. A pilot study using human participants (N = 7) who were naïve to the purpose of the present study and the experimental task revealed that an increase in DoS improved the intensity of goal directedness (see Supplementary information for the pilot study). However, their evaluation of stimuli with the highest correlations (1.0 DoS) was significantly low. These results were consistent with those of previous studies^19^,^20^,^21^,^22^ and supported the argument that completely synchronous motion reduces animacy impression. Therefore, we defined the movie clips with the highest ratings as Chasing scenarios (0.8 and 0.9 DoS) and those with the lowest ratings as Random scenarios (0.0 DoS).

Two adult female Japanese macaques (AR2088 and AR2387) and eight adult humans participated in Experiments 1 and 2. Another adult female monkey (AR2212) was additionally employed in Experiment 2. Participants were trained in operant conditioning settings adapted for each species (see Methods). The stimuli were movie clips, each containing two geometric moving particles with constant speed. We produced movies with either Chasing or Random sequences (see Supplementary information for details and an example of stimuli; see Videos 1 and 2).

The monkeys and human participants were trained to discriminate between Chasing and Random scenarios using a go/no-go procedure. In this task, participants were required to respond only to Chasing scenarios. Following the training phase, two experiments were conducted using a probe test. Experiment 1 sought to determine whether participants completed the task by focusing on correlations. Stimuli with various levels of DoS were used (six steps from 0.0 to 1.0 DoS conditions). Experiment 2 sought to determine whether participants solved the task using the proximity of moving particles. The distance between the particles was identical in both Control and Chasing scenarios, but each particle moved haphazardly in Control scenarios.

## Results and Discussion

### Experiment 1

#### Training

One monkey (AR2088) completed 30 sessions, and the other (AR2387) completed 23 sessions to fulfil the correct-response criterion. Human participants completed two consecutive training sessions, and their performance was maintained with a correct-response ratio of over 80% (Wilcoxon signed-rank test: mean = 90.63%, *p* < 0.01, effect size *r* = 0.73, 95% confidence interval; CI = [90.00, 97.50]).

#### Testing

In the baseline trials, Wilcoxon signed-rank tests revealed that the monkeys achieved a correct-response ratio of over 80% (AR2088: mean = 86.80%, *p* = 0.01, *r* = 0.78, CI = [82.50, 90.59]; AR2387: mean = 89.10%, *p* < 0.01, *r* = 0.87, CI = [85.00, 93.00]).

**Figure 1:**
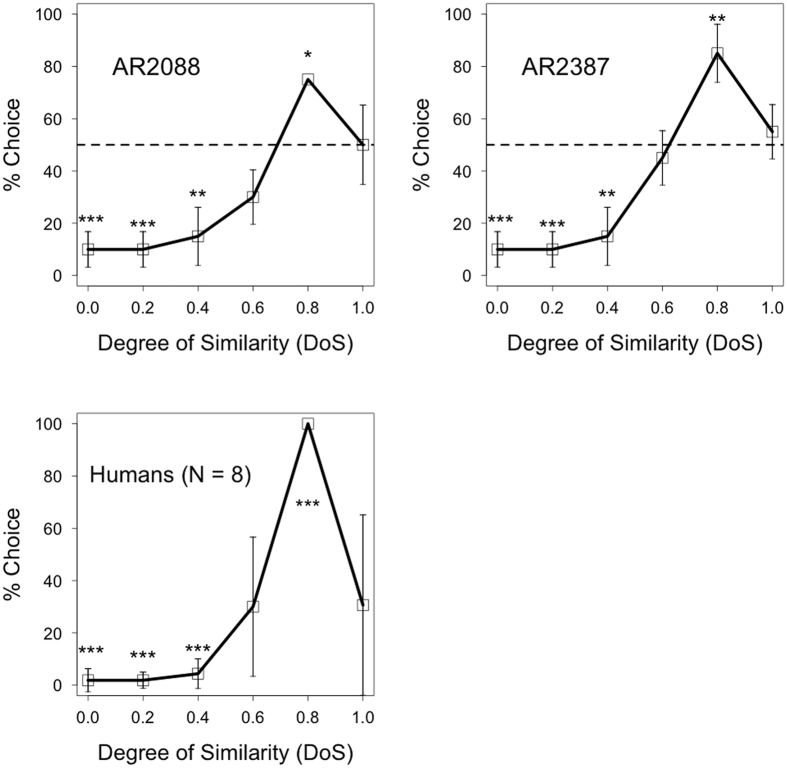
Results of the test sessions in Experiment 1. The upper panel shows performance for each monkey; the lower shows performance for human participants. The solid lines indicate the percentage of choice responses for each test stimulus. The dotted line represents chance level (50%). The asterisks indicate significant departures from chance, calculated using two-tailed binomial tests for monkeys and Haberman’s residual analyses for humans (*p < 0.05; **p < 0.01; ***p < 0.001). Error bars represent the ±95% confidence interval for the mean.

Figure 1 shows the percentages of choice responses to the test stimuli in monkeys and humans. The monkeys responded at significantly lower than the 50% chance level in the 0.0, 0.2, and 0.4 DoS conditions (two-tailed binomial tests; *p* < 0.01) and higher than chance in the 0.8 DoS condition (*p* < 0.05). The percentages of choice responses in the 0.6 and 1.0 DoS conditions were identical to those of chance in the monkeys (*p* > 0.10). We analysed human performances using Haberman’s residual analysis to estimate the significant differences in their response numbers from expected response numbers in each condition. The results suggest that the number of choice responses in the 0.0, 0.2, and 0.4 DoS conditions were significantly lower than chance levels (residuals = −42, −42, and −38, *p* < 0.001, respectively), and they chose the stimulus with 0.8 DoS at the rate of 100% (residual = 115, *p* < 0.01). The number of choice responses in the 0.6 and 1.0 DoS conditions were identical to those of the expected chance level (residuals = 3 and 4, *p* = 0.56 and 0.44, respectively). The two species exhibited similar response trends, and the results seemed to reflect the evaluations made by human participants in the pilot study. Participants discriminated chasing sequences not only from other motion types with lower correlations between trajectories but also from those with higher correlations.

### Experiment 2

#### Training

AR2212 completed 61 sessions to fulfil the correct-response criterion.

#### Testing

In the baseline trials, Wilcoxon signed-rank tests revealed that AR2088 achieved a correct-response ratio of over 80% (mean = 93.20%, *p* < 0.01, *r* = 0.87, CI = [91.50, 94.50]), AR2387’s performance was identical to that required by the correct-response criterion (mean = 79.90%, *r* = 0.00, *p* = 1.00, CI = [75.00, 84.00]), and AR2212’s performance was higher than the criterion (mean = 88.90%, *p* < 0.01, *r* = 0.84, CI = [83.00, 92.50]).

**Figure 2:**
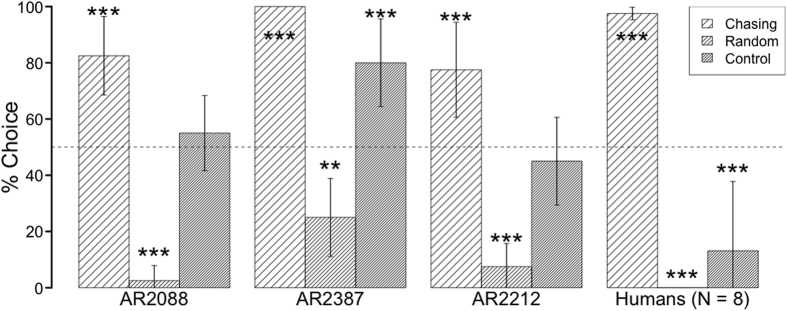
Results of the test sessions in Experiment 2. The right panel shows performance for human participants; the others show performance for each monkey. The bars indicate the percentage of choice responses for each test stimulus. The dotted line represents chance level (50%). The asterisks indicate significant departures from chance, calculated using two-tailed binomial tests for monkeys and Haberman’s residual analyses for humans (**p < 0.01; ***p < 0.001). Error bars represent the ±95% confidence interval for the mean.

Figure 2 shows the mean percentages of choice responses in the test trials for all participants. Participants chose Chasing sequence test stimuli at a higher rate than the chance level and Random sequences at a lower rate than that of chance (binomial tests for monkeys, *p* < 0.001; Haberman’s residual analyses for humans, residuals = 97 and −59, *p* < 0.001, respectively). One monkey, AR2088, responded to Control sequences at the chance level while the other chose these stimuli at a higher rate than that of chance; humans chose these stimuli at a lower rate than that of chance (residual = −38, *p* < 0.001). However, we found a significant effect of condition in the monkeys (one-way Friedman tests, AR2088: *X*^2^ = 7.60, d.f. = 2, *p* = 0.02; AR2387: *X*^2^ = 17.89, d.f. = 2, *p* < 0.001; AR2212: *X*^2^ = 15.74, d.f. = 2, *p* < 0.001). Subsequent multiple comparisons using the Steel-Dwass method revealed that they chose Chasing scenarios more frequently than Random (*p* < 0.001) and Control (AR2088: *p* = 0.03; AR2387: *p* = 0.01; AR2212: *p* = 0.02) sequences. They responded to Control scenarios more frequently than Random sequences (AR2088 and AR2212: *p* < 0.001; AR2387: *p* = 0.01). For human participants, a one-way repeated measures Friedman test showed a significant effect of the stimulus condition (*X*^2^ = 14.89, d.f. = 2, *p* < 0.001). Subsequent multiple comparisons by Steel-Dwass method showed that they chose Chasing sequences more frequently than Random (*p* < 0.001) and Control (*p* = 0.002) sequences; however, choice frequencies did not significantly differ from Random and Control sequences (*p* = 0.15). Participants discriminated between Chasing and Control stimuli, indicating that the two species did not use constant closeness between the particles as the main cue to complete the task. This suggests that they integrated motion direction and target locations perceptually rather than using a simple spatiotemporal contingency between particles.

### General Discussion

The results of the present series of experiments suggest that the Japanese macaques perceived goal directedness using motion information from geometric particles rather than detecting partial physical properties. High correlation and proximity are typical characteristics of chasing events, but our data suggested that the observers were not restricted to identifying each property with the discriminative cue. The human visual system is specifically designed to extract self-produced and goal-directed motion patterns from the environment to ensure that we attend to them quickly and automatically^9^,^11^,^19^,^20^,^22^,^24^. Our findings suggest that this cognitive strategy is not unique to humans. For natural scenes, some behaviour with important adaptive consequences can be inferred from whole-body motion^25^. Researchers have argued that humans from early infancy have schemas for encoding each behavioural category into motion events^8^,^26^. Identifying whole-body motions by agents with corresponding categories would have evolutionary advantages^25^. Our current finding supports this idea, and this cognitive phenotype may not only be there in primates, but also in other social organisms.

To date, the looking-time paradigm has succeeded in revealing discriminative responses to the goal-directed action of inanimate agents in humans (e.g., a human infant study)^7^ and chimpanzees^14^,^15^. The findings in a study of squirrel monkeys^18^ and ours suggest that operant conditioning techniques can also extract the sensitivities to goal of agents without any bodily features from the animals. These techniques may allow us to compare various species, which would not show inherent gaze toward goal–agent associations.

We must note that our data still has some potential limitations to conclude that Japanese macaques have human-like cognitive capacity. First, operant conditioning techniques are often used to elicit unnatural behaviours for the animal, and this may lead to results without ecological validity. In this study, we trained the monkeys to discriminate between two displays, and they were then allowed to select various strategies to perform. In this case, the monkeys’ choices must rely on their ecological background and a series of our probe tests could assess such spontaneous behaviours. One of the supportive assessments elucidates the correlation between their performances and natural behavioural repertoires^25^. In addition to that, cross-species comparisons may also be helpful. The second important limitation is the small sample size. Long-term training often reduces the number of subjects; thus, the species-level conclusion would be debatable. Our study was designed to test each individual carefully to obtain an evidence to reject the null hypothesis, that is, Japanese macaques do not attribute a goal to inanimate objects.

The domain-specific knowledge of motion information used to attribute others’ goals in humans seems to be adapted to various primates, and the evolution of this capacity could contribute to the emergence of our reasoning other’s mind.

## Methods

### Experiment 1

#### Participants

Two adult female Japanese macaques, AR2088 (9 years old) and AR2387 (4 years old), and eight adult humans (five males and three females; mean age = 20.25 years) participated in the study. The monkeys were housed alone; one per cage, but they were allowed visual and auditory communication with other monkeys in the same room. The monkeys were not deprived of food; however, they were given the food only after all the daily sessions were completed. Prior to this study, the monkeys and the human participants were never exposed to the experimental tasks used in this study. All experiments involving human participants were carried out in accordance with the *Guide for Experimentation with Humans* provided by the Primate Research Institute, Kyoto University (KUPRI). The experimental protocol was approved by the Human Research Ethics Committee of KUPRI, and written informed consent was obtained from all participants. All experiments involving monkeys complied with the 3^rd^ edition of the *Guide for the Care and Use of Laboratory Primates* issued by KUPRI in 2010 and were approved by the Ethics Committee of KUPRI.

#### Apparatus

Each monkey was trained in an operant conditioning box (45 × 45 × 60 cm) located in a dark, sound-attenuated room with a continuous white noise. The front wall of the box had an opening. A 21.5-inch touch-sensitive LCD screen (1920 × 1080 pixel display resolution), used for stimulus presentation, was mounted on the front panel of the box. A food dispenser delivered a piece of sweet potato through a tube into a food cup mounted on the bottom of the front wall of the box. The room was illuminated during each session by a ceiling-mounted house light. USB I/O interfaces controlled the house light and feeder, and Visual C# (Microsoft, 2010) generated the stimuli and controlled the experimental procedure. The apparatus used in the human experiment was identical to that used in the pilot study (see Supplementary information).

#### Stimuli

We used two types of training stimulus: Chasing and Random motion patterns generated by an identical algorithm from the pilot study. With the exception of motion paths, these two types of movie clip were the same as those used in the pilot study. The DoS between the trajectories of two moving objects was kept between 0.90 and 0.95 in Chasing scenarios and between 0.00 and 0.05 in Random scenarios. While testing, we introduced new movie clips with new trajectories, in which the DoS was systematically altered for the Chasing and Random motion types used in training. We added six types of movie with 0.0, 0.2, 0.4, 0.6, 0.8, and 1.0 DoS. The motion paths for the stimuli in each condition were identical to those used in the pilot study; therefore, each condition constituted 5 movie clips.

#### Procedure

##### Training

We trained two monkeys (AR2088 and AR2387) and eight adult humans using a go/no-go discrimination procedure. To provide a response, the monkeys were required to touch a visual cue on the monitor, and the humans used a keypad. The humans participated in this experiment for one day only. Prior to the experiment, human participants were instructed to solve a task of trial-and error learning based on the sound feedback mentioned below. This type of experimental setting for human participants is widely accepted in comparative researches^27^,^28^.

Each trial began with a square-shaped white start key (5 × 5 cm) at the centre of the display. It disappeared after it was touched (key press for humans), and immediately one of the two types of stimulus (Chasing or Random) started playing. Each trial lasted for a maximum of 10 s (seconds), and consisted of two parts; the first was a 5-s period and the second part was between 0–5-s. Responses during the first 5-s period were disregarded. After this initial 5-s period, an orange coloured frame appeared around the stimulus and the responses were monitored. If the participant responded during a Chasing trial or did not respond during a Random trial, the display turned black, followed by reinforcement (food) and a holo-holo sound (human participants received the sound only), and an inter-trial interval (ITI) began. If the participant responded during a Random trial or made no response during a Chasing trial, a buzzer sound was made and the display turned dark for 6 s as a time-out (the house light also went out during the time-out period in monkey experiments). Following the time-out, the participant completed one or more correction trials until the correct response was made. For the monkeys, if the number of correct trials was lower than the previous day, the time-out duration was extended from 6 s to a maximum of 30 s. After the first 5 s had passed, the trials were terminated either at the end of the maximum duration (i.e., 10 s) or with the participant’s response. The ITI lasted for at least 3 s; however, if the participant responded during a Chasing trial or made no response during a Random trial, the subsequent ITI was temporarily extended by the time difference between the maximum trial duration and the reaction time. For the monkeys, each daily training session contained two blocks of 50 trials. For humans, at least two training sessions were required, and each session contained 20 trials with two presentations of each movie clip. This asymmetric design between the species was employed because we predicted that our task would be quite easy for humans. As expected, the results of the human training sessions indicate that they met the same learning criterion as monkeys (see the result section of Experiment 1). Each stimulus was presented in a pseudo-random sequence to ensure that a type of stimuli (Chasing or Random) did not appear three or more times consecutively. The training continued until the monkeys reached a correct-response criterion stipulating that at least 80% of choices should be correct over two consecutive sessions. The monkeys were tested immediately after reaching this criterion. Note that the correction trials and occasional incomplete sessions were not analysed.

##### Testing

The monkeys were presented with 112 trials per test session (10 sessions in total). The test sessions included 50 presentations of the two training stimuli (baseline trials) and 12 presentations of each test stimulus (test trials). In the baseline trials, the procedures were identical to those of the training trials. In the test trials, the trial lasted for a maximum of 10 s, and the monkeys did not receive food rewards or penalties. Each testing condition included 5 movie clips, and each clip was presented twice within a session. Each movie clip was employed in 2 sessions, thus, a total of 20 trials were involved in each testing condition. The trials in each session were presented in a pseudo-random order, but the first 10 trials did not include test trials. The response ratio was calculated using the number of responses in each condition.

There were 120 trials for human participants. Each condition included 20 trials consisting of five different movie clips presented four times. Participants were not given feedback for their responses. Before the test started, participants were instructed to perform a task based on what they had learned during the training session.

### Experiment 2

#### Participants

The monkeys (AR2088 and AR2387) and human participants involved in Experiment 1 also participated in this experiment. Additionally, we employed another adult female monkey (AR2212, 8 years old) to support our argument. The third monkey was also naïve to the experimental tasks in this study.

#### Apparatus

The apparatus used for monkeys and humans was identical to that used in Experiment 1.

#### Stimuli

The types of training stimuli used in the experiment involving monkeys were identical to those of Experiment 1 (i.e., Chasing and Random scenarios). In this experiment, we used new Chasing and Random sequences involving different trajectories to those used in Experiment 1 (5 clips for each stimulus condition). During testing, we used additional new Chasing (0.9 DoS) and Random (0.0 DoS) sequence clips as probe stimuli to determine whether the monkeys memorized specific movie clips. In addition to that, we introduced another stimulus ‘Control’ (see Video 3) to determine whether the proximity of two geometric particles was used as a discriminative cue for Chasing scenario discrimination. Training stimuli for monkeys and test stimuli for both species were produced by the computer program used in the pilot study and Experiment 1. Movie clips in ‘Control’ condition were produced by the algorithm used to generate Random sequences; however, we used those clips where the distance of particles remained closer throughout the sequence. A two-tailed *t* test confirmed that the distances between moving objects were identical to those in Chasing and Control test sequences (Chasing: mean = 1.46 cm, SD = 0.03; Control: mean = 1.50 cm, SD = 0.04; *t*_8_ = 1.38, *p* = 0.21, *r* = 0.44, CI = [−0.72, 2.85]).

#### Procedure

The procedure was identical to that of Experiment 1 for all participants. Prior to the test phase in the experiment involving monkeys, we conducted training sessions that followed an identical procedure to that of Experiment 1. One monkey (AR2212) was trained until she met the criterion while two monkeys (AR2088 and AR2387) completed at least two training sessions. We then confirmed that the monkeys had fulfilled the correct-response criterion. The test sessions for monkeys included 100 baseline (50 Chasing and 50 Random), and 12 test trials per session. The test trials constituted three conditions, each involving four trials showing one movie clip. We conducted 10 test sessions, showing five movie clips in each condition; therefore, each test clip was presented twice. Training sessions were not completed by human subjects. The second experiment involving humans was introduced soon after they had completed Experiment 1. Prior to the experiment, they were instructed to perform a task based on the training sessions. One test session contained three conditions, each involving 20 trials. Four movie clips of each condition were shown five times within a session. The participants were not given feedback during the experiment.

#### Statistical analysis

The data obtained from the two experiments were analysed using non-parametric tests. The data from each condition for human participants were assessed for normality using Shapiro-Wilk tests. We also used non-parametric tests for subsequent statistical analyses of human group data. The performances during the training sessions for both the species and the baseline trials for monkeys were estimated by Wilcoxon signed-rank tests. The significant differences of the number of responses in testing condition were estimated by two-tailed binomial tests for the monkeys. For the human data, we used Haberman’s residual analysis for both the experiments. In the Experiment 2, the effect of conditions and its post-hoc analyses were performed by Friedman tests and Steel-Dwass tests for both the species.

## Additional Information

**How to cite this article**: Atsumi, T. *et al*. Goal attribution to inanimate moving objects by Japanese macaques (*Macaca fuscata*). *Sci. Rep.* **7**, 40033; doi: 10.1038/srep40033 (2017).

**Publisher's note:** Springer Nature remains neutral with regard to jurisdictional claims in published maps and institutional affiliations.

## Supplementary Material

Supplementary Information

Supplementary video 1

Supplementary video 2

Supplementary video 3
