# Supplementary material for: Goal attribution to inanimate moving objects by Japanese macaques (Macaca fuscata)
Source: Sci Rep. 2017 Jan 5;7:40033. doi: 10.1038/srep40033 (PMC5215463; doi:10.1038/srep40033)
Supplement: Supplementary Information [file srep40033-s1.pdf]

Supplementary information

Goal attribution to inanimate moving objects by Japanese macaques (*Macaca fuscata*)

Takeshi Atsumi[1,*], Hiroki Koda[2], Nobuo Masataka[2]

[1]Research Institute of National Rehabilitation Center for Persons with Disabilities, Tokorozawa, Saitama 359-8555, Japan

[2]Primate Research Institute, Kyoto University, Inuyama, Aichi 484-8506, Japan

[*]e-mail: atsumi-takeshi@rehab.go.jp

Pilot study

Supplementary Methods

Participants. Seven adult humans (one man and six women) with normal or corrected-to-normal vision participated in the study. This experiment was carried out in accordance with the Guide for Experimentation with Humans by the Primate Research Institute, Kyoto University (KUPRI). The experimental protocol was approved by the Human Research Ethics Committee of KUPRI, and written informed consent was obtained from all participants.

Apparatus. Stimuli were presented on a laptop computer screen (FM-V Biblo, Fujitsu, Japan, 1,024 × 768 pixel display resolution). Subjects responded using a keypad.

Stimuli. The stimuli were movie clips, each containing two round black particles (5 mm in diameter) that moved on a white circle (14 cm wide and 14 cm high). At the beginning of each clip, the particles were displayed at the centre of the circle, with a distance of 1.67 cm between them. After the particles appeared, they immediately began moving at a constant speed of 5 cm/s. The movies played at a rate of 30 frames/s. We made two types of movie for the discrimination task in the subsequent comparative study (see Experiments 1 and 2 mentioned below): one depicted Chasing stimuli and the other depicted Random stimuli. In Chasing movie, one particle was assigned as a runner and the other as a chaser (see Video 1 for a clip of this animation). The runner moved randomly but appeared to avoid the chaser, in that its motion direction was randomly directed towards the spaces that were not occupied by the chaser. The chaser continuously moved towards the runner’s position, indicating that it was attempting to become closer to the runner. Random stimulus movie contained two randomly moving particles (see also Video 2). In the test sessions, we introduced new movie clips with new

trajectories. In Random stimuli, particles moved haphazardly and randomly changed direction within a 360° window every 0.33 s in both the training and testing sessions. The chasers in the chasing stimuli also moved randomly and kept the same timing of direction change as in the Random stimuli. All random motion was constrained by the specification that the two particles would neither come into contact with each other nor overlap.

Chasing and Random movie clip prototypes were generated using an algorithm of our computer program, and the scenarios were defined arbitrarily. Therefore, prior to initiation of the comparative study, it was necessary to examine the validity of our definitions of the stimulus types. A high degree of correlation between interacting agents is often observed in social events represented by motion cues[1–4]. To examine whether the correlation between the trajectories of two particles affected the perception of chasing events, we controlled the similarities in the particle trajectories based on correlations between the object coordinates at each frame of the sequence. We calculated the degree of similarity (DoS) between two trajectories using Pearson's correlation coefficients. First, we calculated the correlation coefficients between the horizontal and vertical positions of each particle during the entire sequence; the average of the two correlation coefficients was defined as the DoS between the trajectories of two moving particles. The trajectories in the arbitrarily defined Chasing stimuli showed a high DoS (pilot study mean: 0.93, standard deviation: 0.02), and those in Random scenarios were completely different (mean: 0.00, SD: 0.02). To evaluate the relationship between DoS and discrimination performance, we systematically altered the similarity in Chasing sequences and used the modified versions of the movies. We created Chasing scenarios in which the DoS was 0.0, 0.1…0.8, 0.9, or 1.0 (steps of 0.1, 11 conditions). In the

maximal DoS condition (1.0), the two trajectories were completely identical, and this was named 'Clone' stimulus, as used in a previous study[5]. In this movie clip, the trajectories of the two particles were always the same (i.e., the motion of one particle seemed to copy that of the other), and the distance between them was stable.

Procedure. Each condition included 5 movie clips, which were presented 5 times within a session. Participants watched one movie in every trial. The trial began with participants pressing any key; thereafter, a stimulus immediately began playing and lasted for 5 s. When the movie finished, participants reported the intensity of goal-directedness in the sequence; one particle moved to become closer to the other in the sequence[6]. They rated each stimulus on a 7-point scale (1 = definitely not goal-directed, 7 = definitely goal-directed). Following the subject's response, an inter-trial interval (ITI) began and lasted for 3 s. Mean ratings were calculated for all participants in each condition, and goal-directedness ratings were analysed for each condition.

Supplementary Results and Discussion

The results showed that goal-directedness ratings were positively correlated with trajectory similarity (Spearman's rank correlation, $\rho = 0.60$, $p < 0.001$). The ratings increased monotonically with increasing trajectory similarity from the 0.0 DoS condition to the 0.9 DoS condition but dropped sharply in the 1.0 DoS condition (i.e., Clone). We assessed the data from each subject for violation of normality with the Shapiro-Wilk test. Accordingly, we applied non-parametric tests for subsequent statistical analyses. A one-way Friedman test found a significant effect of DoS ($X^2 = 40.43$, d.f. = 6, $p < 0.001$). Subsequent pairwise comparisons with Mann-Whitney test showed a significant difference in ratings between the 0.8/0.9 and 1.0 DoS conditions ($p < 0.05$, effect size; r

> 0.81). The 0.8 and 0.9 DoS conditions did not differ significantly ($p = 0.61$, $r = 0.19$); therefore, these two stimuli were considered goal-directed motion patterns. These results indicated that the goal-directedness of Chasing scenarios could not be described using only the correlation between the trajectories of moving objects. Greater randomness between the two objects weakened perceived goal-directedness; however, the result suggested that strong constraint between the two moving objects was also able to reduce this impression. In Clone scenarios (1.0 DoS), the motion of the two objects was identical, while each object in Chasing scenarios (0.8 or 0.9 DoS) engaged in a different manner of motion (i.e. a chaser and a runner). This type of completely synchronous motion event led to weakly perceived intention and animacy in humans[7]. Our results support previous findings.

In the testing involving Japanese macaques (*Macaca fuscata*), we selected stimuli with various ranges of correlation between the trajectories of the two particles to examine whether the monkeys differentiated between Chasing stimuli and those involving other types of motion.

References

1. Gao, T., Newman, G. E. & Scholl, B. J. The psychophysics of chasing: a case study in the perception of animacy. *Cognit Psychol* **59**, 154–179 (2009).
2. Gao, T. & Scholl, B. J. Chasing vs. stalking: interrupting the perception of animacy. *J Exp Psychol Hum Percept Perform* **37**, 669–684 (2011).
3. Gao, T., Scholl, B. J. & McCarthy, G. Dissociating the detection of intentionality from animacy in the right posterior superior temporal sulcus. *J Neurosci* **32**, 14276–14280 (2012).
4. Schultz, J., Friston, K. J., O'Doherty, J., Wolpert, D. M. & Frith, C. D. Activation in posterior superior temporal sulcus parallels parameter inducing the percept of animacy. *Neuron* **45**, 625–635 (2005).
5. Atsumi, T. & Nagasaka, Y. Perception of chasing in squirrel monkeys (*Saimiri sciureus*). *Anim Cogn* **18**, 1243–1253 (2015).
6. Opfer, J. E. Identifying living and sentient kinds from dynamic information: the case of goal-directed versus aimless autonomous movement in conceptual change. *Cognition* **86**, 97–122 (2002).
7. Takahashi, K. & Watanabe, K. Synchronous motion modulates animacy perception. *J Vis* **15**, 1–17 (2015).
